# Medical innovations to maintain the function in patients with chronic PJI for whom explantation is not desirable: a pathophysiology-, multidisciplinary-, and experience-based approach

**DOI:** 10.1051/sicotj/2020021

**Published:** 2020-07-07

**Authors:** Tristan Ferry, Cécile Batailler, Sophie Brosset, Camille Kolenda, Sylvain Goutelle, Elliot Sappey-Marinier, Jérôme Josse, Frédéric Laurent, Sébastien Lustig

**Affiliations:** 1 Service des Maladies Infectieuses et Tropicales, Hospices Civils de Lyon, Hôpital de la Croix-Rousse 93 Grande Rue de la Croix-Rousse 69004 Lyon France; 2 Université Claude Bernard Lyon 1 69100 Villeurbanne France; 3 Centre Interrégional de Référence Pour la Prise en Charge des Infections Ostéo-Articulaires Complexes (CRIOAc Lyon), Hôpital de la Croix-Rousse 93 Grande Rue de la Croix-Rousse 69004 Lyon France; 4 CIRI – Centre International de Recherche en Infectiologie, Inserm, U1111, Université Claude Bernard Lyon 1, CNRS, UMR5308, Ecole Normale Supérieure de Lyon, Univ. Lyon 69007 Lyon France; 5 Service de Chirurgie Orthopédique, Hôpital de la Croix-Rousse 93 Grande Rue de la Croix-Rousse 69004 Lyon France; 6 Service de Chirurgie Plastique et Reconstructrice, Hôpital de la Croix-Rousse 93 Grande Rue de la Croix-Rousse 69004 Lyon France; 7 Institut des Agents Infectieux, Laboratoire de Bactériologie, Centre National de Référence des Staphylocoques, Hôpital de la Croix-Rousse 93 Grande Rue de la Croix-Rousse 69004 Lyon France; 8 Service de Pharmacie, Hospices Civils de Lyon, Groupement Hospitalier Nord, Hôpital Pierre Garraud 136 Rue du Commandant Charcot 69005 Lyon France; 9 UMR CNRS 5558, Laboratoire de Biométrie et Biologie Evolutive Villeurbanne France

**Keywords:** PJI, Innovations, Bacteriophages, Phage therapy, Lysins, Suppressive antimicrobial therapy, Antibiotics

## Abstract

*Introduction*: PJI is the most dramatic complication after joint arthroplasty. In patients with chronic infection, prosthesis exchange is in theory the rule. However, this surgical approach is sometimes not desirable especially in elderly patients with multiple comorbidities, as it could be associated with a dramatic loss of function, reduction of the bone stock, fracture, or peroperative death. We propose here to report different approaches that can help to maintain the function in such patients based on a pathophysiology-, multidisciplinary-, and an experience-based approach. *Methods*: We describe the different points that are needed to treat such patients: (i) the multidisciplinary care management; (ii) understanding the mechanism of bacterial persistence; (iii) optimization of the conservative surgical approach; (iv) use of suppressive antimicrobial therapy (SAT); (v) implementation of innovative agents that could be used locally to target the biofilm. *Results*: In France, a nation-wide network called CRIOAc has been created and funded by the French Health ministry to manage complex bone and joint infection. Based on the understanding of the complex pathophysiology of PJI, it seems to be feasible to propose conservative surgical treatment such as “debridement antibiotics and implant retention” (with or without soft-tissue coverage) followed by SAT to control the disease progression. Finally, there is a rational for the use of particular agents that have the ability to target the bacteria embedded in biofilm such as bacteriophages and phage lysins. *Discussion*: This multistep approach is probably a key determinant to propose innovative management in patients with complex PJI, to improve the outcome. *Conclusion*: Conservative treatment has a high potential in patients with chronic PJI for whom explantation is not desirable. The next step will be to evaluate such practices in nation-wide clinical trials.

## Introduction

Prosthetic-joint infection (PJI) is the most dramatic complication after joint arthroplasty [1–3]. In patients with acute infection, the recommended treatment is open debridement antibiotics and implant retention (DAIR) with exchange of the mobile polyethylene part, followed by antibiotics usually for a total duration of 12 weeks [2, 4–6]. In patients with chronic PJI, the recommended strategy is to exchange the prosthesis, in a 1-stage or a 2-stage procedure, to mechanically eradicate the biofilm that is a community of bacteria that definitely adhere to the implant. However, prosthesis explantation is sometimes not feasible, especially for the knee location in elderly patients with multiple comorbidities for whom explantation could be associated with a dramatic loss of function, reduction of the bone stock, fracture, or peroperative death [2, 4–6]. Indeed, explantation without reimplantation, also called resection arthroplasty, or Gilderstone procedure, is possible for the hip but not recommended for the knee, as the functional consequences are not acceptable, even if some authors reported exceptional favorable outcomes [7–10]. Moreover, reimplantation using silver-coated arthrodesis could be more complex as expected and transfemoral amputation is associated with a poor functional outcome [11, 12]. Which is why open DAIR is sometimes proposed for such patients, especially in patients without prosthesis loosening, but the risk of relapse is particularly high due to the bacterial persistence in biofilm at the implant surface [2, 4–6].

As it is impossible to eradicate the bacteria embedded in the biofilm in patients with chronic PJI for whom a conservative treatment is performed, a suppressive antibiotic treatment (SAT) is usually proposed to these patients following an open DAIR procedure [2, 4, 5]. SAT consists in daily oral intake of active antibiotic to suppress the infection i.e. to alleviate the symptoms and to prevent the progression of the infection without hope for eradication [2, 4, 5]. In cohort studies, the outcome (i.e. the control of the clinical signs of infection) is favorable in 30–70% of patients, depending on the patient profile, the pathogen involved, the drug used and the duration of follow-up [13–21]. In this context, the use of new adjuvant therapies that target locally the bacterial biofilm is of great interest as it may increase the probability of SAT to control the disease.

We propose here to report the different medical approaches that can help to maintain the function in patients with chronic PJI for whom explantation is not desirable, based on a pathophysiology-, multidisciplinary-, and experience-based approach. This review is mainly based on our experience as regional reference center for the management of complex bone and joint infection (CRIOAc Lyon; http://www.crioac-lyon.fr) that belongs to a network set up by the French health ministry to promote innovation and improve the patient care.

## A nation-wide dedicated network for the management of complex BJI

**Figure 1:**
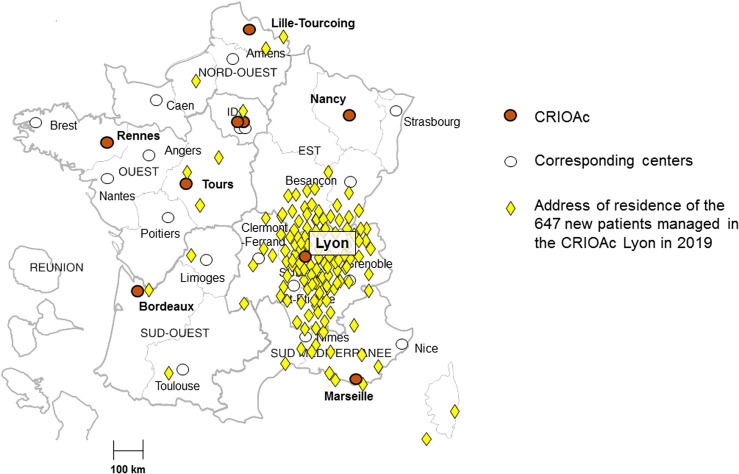
The CRIOAc network in France labeled by the French Health Ministry (*Direction Générale de l’Offre de Soin*s): reference centers appear in orange rounds, associated centers in white rounds and the address of residence of the 647 new patients managed in the CRIOAc Lyon in 2019 in yellow diamond (adapted from the maps available here: http://www.crioac-lyon.fr/origine-patients.html).

Some BJI such as fracture-related infections (FRI) and PJI are some of the most difficult-to-treat bacterial diseases [1, 22, 23]. Moreover, there is a considerable functional challenge for these patients, as aggressive surgery, sometimes required to treat the infection, may considerably alter the function especially if the reconstruction required few used and costly implants with low experience concerning their survival during the follow-up [11, 23, 24]. It is important to note that these BJI also have a huge economic impact for the hospital and the health care system, especially in case of hospital readmission, that is usually in link with the reconstruction, a bacterial persistence or the occurrence of a new infection [25–27]. Of note, a new infection due to multidrug-resistant bacteria increases considerably the cost. In the USA, the annual cost to hospitals of revision surgery for infection increased from $320 million in 2001 to $566 million in 2009, and was projected to exceed $1.62 billion by 2020 [28]. Facing the medical challenges and the cost of such infections, the “*Direction Générale de l’Offre de Soins*” (DGOS; French Health Ministry) decided to create and fund in 2008 a dedicated nation-wide network of reference centers for the management of complex BJI called “*Centre de Référence des Infections Ostéoarticulaires complexes*” (CRIOAc) [29]. This network was founded in collaboration with six French health-care societies representing physicians, scientists and other health-care professionals involved in the management of BJI. The budget is of ~€100,000 per CRIOAc, for the purpose to facilitate performance of multidisciplinary meetings, events organization, information, and inter-regional coordination. The activity-dependent budget is based on the “PMSI” (*Programme de médicalisation des systèmes d’information*) data-base that is a French national hospital discharge database. A specific code was created and used for each hospital stay for complex BJI. Using this code, for patients for whom a complex BJI was discussed in multidisciplinary meeting, the hospital receives an extra 12% funding for the surgical hospital stay from the national health insurance system. Criteria for complex BJI are the following: (i) host criteria: patient with severe comorbidity limiting treatment options, or patient with severe allergy; (ii) microbiological criteria: difficult-to-treat micro-organism(s) with or without multidrug resistance; (iii) surgical criteria: BJI requiring bone resection and bone and/or soft-tissue reconstruction; and (iv) relapse of a previous episode of BJI. For use by all of the labeled centers, a dedicated secure national online information system was designed to collect the data during multidisciplinary meetings such as medical history, clinical characteristics, the type of BJI, pathogen involved and finally the surgery and antibiotics proposed. The online information system has as final objectives to: (i) facilitate decision-making during multidisciplinary consultation meetings; (ii) draw up a summary of the patient’s clinical history, the BJI’s complexity status, and decisions taken; (iii) share the medical synthesis in pdf format within the approved structures and with the concerned physicians; (iv) facilitate patient follow-up; (v) produce activity data for assessment of the centers’ missions; and (vi) undertake epidemiological research. Multidisciplinary consultation meetings are the cornerstone of the management of patients with complex BJI in CRIOAc. They have to include orthopedic and plastic surgeons, infectiologists and microbiologists, to discuss each case and to propose a management that seems to be the most relevant, by taking into account each aspect of the disease. Since the set-up of the dedicated online system in 2012, each year ~1000 multidisciplinary consultation meetings are performed in the structures belonging to the network, with ~8000 cases discussed. About 40% are PJI, with a rate of complexity of ~60%. As this networking structure greatly facilitates the synergy between the representatives of each academic disciplines involved, it strongly contributed and still continues to promote research, with clinical trials assessing new diagnostic tools, surgical procedures for a given BJI, specific antibiotics, or overall medical/surgical strategy. A national scientific committee has been created, in collaboration with the DGOS [29]. Epidemiological data will allow feasibility studies and also partnerships with the industry, in the fields of diagnostic, implants, bone cements, bone substitutes, antibiotics and all the various therapeutic alternatives such as bacteriophages. In our center called CRIOAc Lyon, innovative approaches to maintain the function in patients with complex BJI, and especially in patients with PJI, is a clear topic of care and research that was born from the productive interactions between the different local actors. We aim to propose adaptive approaches by propose firstly potentially innovative treatments in patients with dead end clinical situation, as compassionate treatment, and then we will aim to propose national clinical trials, based on this experience. This strategy clearly resulted in receiving multiple requests for patient care inside our region, but also beyond the geographical limit of our region (Figure 1). Clearly, the local structuration in CRIOAc and the national organization of the network is the breeding ground to potentially improve the outcome of patients with BJI, as it facilitated and will facilitate the emergence of clinical research and its direct implementation and evaluation through the network.

## The pathophysiology of chronic PJI

**Figure 2:**
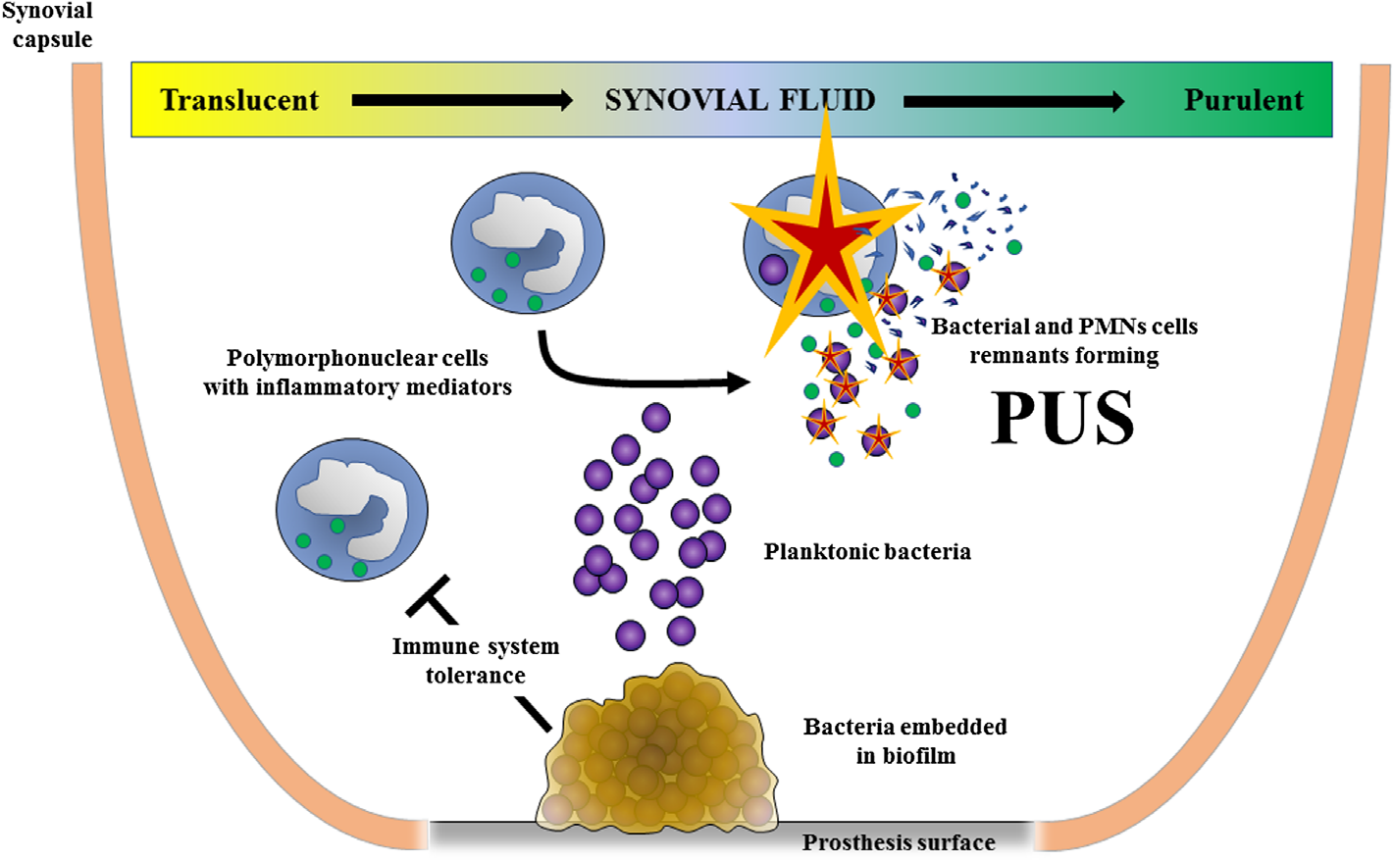
Pathophysiology of prosthetic joint infection with formation of pus due to planktonic bacteria into the joint and formation of bacterial biofilm at the implant surface: Plantonik bacteria are bacteria in “optimal” environmental conditions to growth in the joint liquid, leading to recruitment of polymorphonuclear cells (PMNs), and formation of pus, composed by bacterial and PMNs remnants; Bacteria embedded in biofilm at the implant’s surface, that is inseparable from the implant surface, and tolerant to the immune system.

During PJI, the main way of inoculation of the prosthesis by the bacteria is during the perioperative period, and especially during the time of surgery, despite the fact that several cumulative measures are constantly implemented to prevent and limit this risk. The pathogenesis of PJI involves interactions between the bacteria, the implant, and the host’s immune system. Very few numbers of microbes are needed to infect the prosthesis. Such organisms firstly adhere to the prosthesis surface at the bone-implant interface (stem) and/or into the joint cavity [30]. In the latter, microbes frequently replicate themselves as planktonic bacteria, that are bacteria in “optimal” environmental conditions to growth (i.e., with a lot of nutriments), leading to recruitment of polymorphonuclear cells (PMNs), and clinical signs of septic arthritis (Figure 2). PMNs are major actors that try to control the bacterial multiplication resulting in the formation of pus, that is composed by bacterial and PMNs remnants. At the surface of the implant, most of bacteria have the ability to modify their phenotype and to develop biofilm, after adhering to the surface. Biofilm is defined as a bacterial community which is metabolically heterogeneous and embedded in a self-produced extracellular matrix, a kind of glue that definitively attached the bacterial community to the prosthesis [31, 32]. Once the biofilm is made, it is inseparable from the implant surface, and tolerant to the immune system. It is indeed quite impossible for PMNs to eradicate the biofilm, and other components of the immune system cannot penetrate the biofilm, that mainly contain dormant bacterial cells, with low replication process. Different types of biofilm exist, which form themselves at different speed, depending on the pathogen involved in the PJI. Bacteria can persist for decades in biofilm, and the interaction between the surface of the biofilm and the host cells could lead to prosthesis loosening, by persistent local activation of immune cells [30, 31, 33].

## The conservative surgical approach in patients with PJI

### Debridement antibiotics and implant retention (DAIR)

In patients with chronic PJI, the only way to rule out the biofilm, is to exchange the prosthesis [2, 4, 18]. If it is not desirable for a functional reason, the chosen strategy has to take into account that biofilm eradication (and so the cure) is not possible. The less invasive surgery in patients with PJI and the most “conservative” surgical approach is to perform a “DAIR” procedure, that is most of time performed during arthrotomy (also called open DAIR). The DAIR procedure, described in many papers such as the manuscript of Byren et al. [34] includes firstly the excision of the wound margins followed by removal of necrotic soft tissue, debris, hematoma, or collections of pus (that contain planktonic bacteria) from around the prosthesis. Intra-operative samples are taken at arthrotomy from multiple samples including synovial fluid, synovial tissue, hematoma, and pus in contact to the implant for bacterial culture. The synovial fluid could be also inoculated in blood culture bottles, to facilitate the bacterial culture. Synovectomy and bone samples are usually also performed. Each sample are obtained with separate instruments and placed into separate containers. The prosthesis is assessed for its fixation and its mechanical performance. Any modular prosthesis components has to be exchanged, if possible, especially polyethylene mobile elements. The exposed implant surfaces are irrigated with pulsed lavage with liters of saline to remove adherent planktonic bacteria using dedicated surgical irrigator. Wounds are then potentially closed primarily over drains, which are to be removed at 48 h or when drainage ceased.

### DAIR and soft tissue troubles

**Figure 3:**
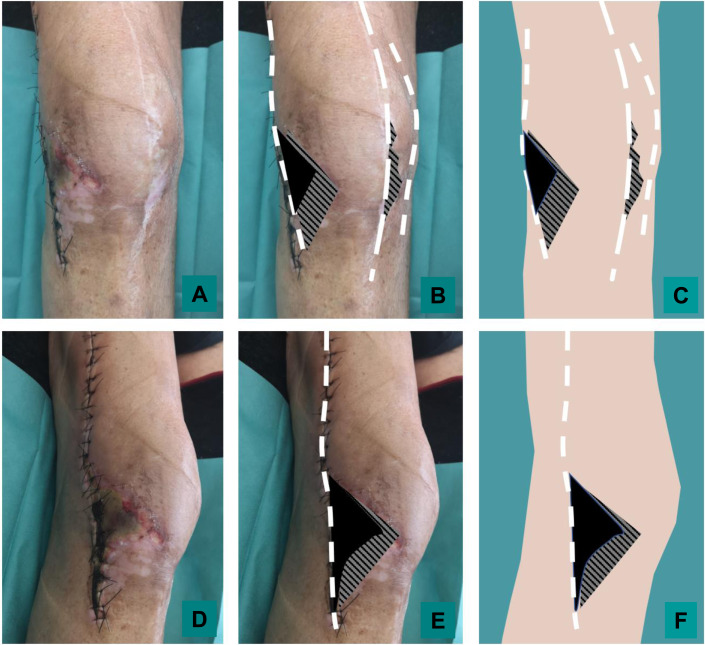
Skin necrosis following knee surgery: local status of a 72-year-old male with skin necrosis 21 days after the revision of a knee arthroplasty (lateral approach). See the multiple scar and white discoloration of the skin in area healed by secondary intention relative to previous superficial necroses. From an anterior (panel A, B and C) and lateral (Panel D, E, F) views, schematic representation of scar (white dotted line), necrosis area (black area) and area healed by secondary intention (hatched area) were added to the photo (panel B and E) and after subtracting the patient’s skin.

**Figure 4:**
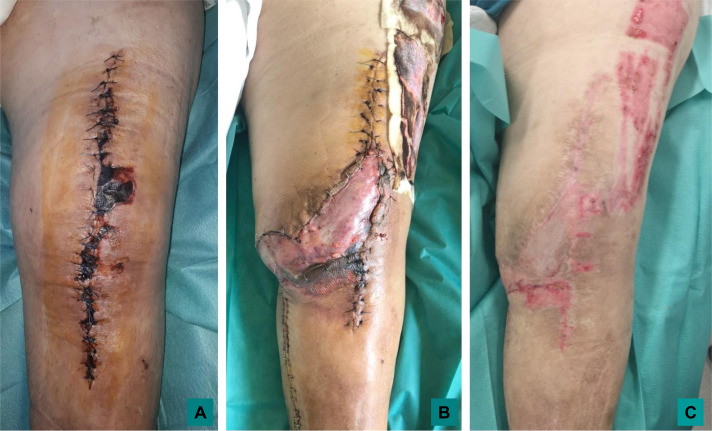
Skin necrosis following DAIR: local status of a 85 year-old-male, experiencing a skin necrosis at 21 days of performance of a left knee arthroplasty (panel A); A DAIR with a medial gastrocnemius flap was performed with satisfactory early results at 14 days (panel B), notice the poor reliability of the skin flap surrounding the anterior tibial tuberosity; and favorable outcome with stable soft tissue coverage at 3 month (panel C).

During DAIR, the surgical approach must at all costs use the previous incision. The multiplication of scars interrupting the vascular network of the knee’s skin, may cause secondary skin necrosis even in young patient. This risk increases when the scars are recent and close from one another (Figure 3). Tension free suture should be performed [35]. In case of prosthetic knee infection, the problem of the soft-tissue envelope should absolutely be considered before DAIR. We can distinguish two different situations: (i) preexisting soft tissue defect or necrosis and (ii) risk of tension suture and incisional dehiscence after DAIR. Wound dehiscence may be a cause or consequence (fistulae) of infection of the underlying prosthesis. In any case, debridement and flap-based reconstruction should be performed during DAIR. Historically, the standard muscle flap for knee coverage has been the medial or lateral gastrocnemius muscle flap [36]; however, with the advent of local and free flap techniques, fasciocutaneous flaps have become popular. Recent studies showed that rates of prosthetic salvage are comparable following muscle or fasciocutaneous flap coverage [37]. Morbidity appears to be less important following fasciocutaneous flap that should be prefer for young patient. In case of impaired vascularization of the lower limb with objective alteration of skin micro vascularization such as brown discoloration of the skin (ochre dermatitis), the reliability of local fascio-cutaneous flap is questionable and muscle flap should be preferred (Figure 4). Free flap coverage should be considered, even for the elderly, if the soft tissue defect is important, or extended to the superior edge of the patella [35]. The soft tissues provided using free flap are abundant, well vascularized, and present an healthy microvascular network, and can be more reliable than local fascio-cutaneous flap. Even in absence of previous soft tissue defect, knee DAIR are at major risk of wound dehiscence, due to local edema and joint effusion and multiple surgical procedure. Soft tissue quality should be evaluated before surgery, preferentially by a plastic surgeon ideally during multidisciplinary meeting. This assessment is based on the evaluation of clinical risk factors of delayed wound healing (tobacco consumption, diabetes, obesity or malnutrition, previous history of delayed wound healing), examination of local tissue flexibility (pinch test) and quality (scar, secondary wound healing area, inflammation, edema) and clinical and paraclinical evaluation of the quality of local arterial and venous vascularization. Flap-based reconstruction is indicated if the risk of suture tension and secondary wound dehiscence appeared to be important to provide durable, well-vascularized coverage that can withstand the dynamic stresses of ambulation and prolonged post-operative edema.

### Arthroscopic DAIR

Arthroscopic DAIR is another way to perform DAIR. It is feasible in patients with prosthetic knee infection, but is considered to have no place in patients with PJI due to: (i) an incomplete debridement (peroperative dislocation is not feasible); (ii) an inability to exchange the polyethylene part of the prosthesis; (iii) a partial reduction of the bacterial load; and (iv) an extremely low success rate, even in the acute setting of PJI [6, 34, 38]. In counterpart, the risk of acquisition of a new infection (also called superinfection) is lower during arthroscopy in comparison with arthrotomy, and it is easy to inject into the joint an innovative antibiofilm preparation such as bacteriophages or bacteriophage-derived lysins (see below), as the joint remained perfectly tight during arthroscopy, in comparison with arthrotomy.

## Suppressive antimicrobial therapy (SAT)

### Oral SAT

Patients with chronic BJI for whom a conservative approach is proposed, could, in theory, not cured, due to the persistence of the bacteria embedded in the biofilm. As these patients are at high risk of relapse, they are candidates for suppressive antibiotic treatment (SAT) [2, 4, 5]. SAT usually followed a “primary” antimicrobial therapy with conventional doses of antibiotics during 6–12 weeks. It consists in daily oral intake of active antibiotic to control the infectious process and prevent the loosening without hope for bacterial eradication. Based on the pathophysiology of the infection, SAT had in theory the potential to keep to bacteria asleep into the biofilm, and limit the new production of planktonic bacteria from the biofilm. IDSA guidelines proposed some oral drugs to be active on most frequent pathogens, but new oral treatment with a safe profile could be interested to be used [2]. In patients without any oral options, we developed the concept of suppressive subcutaneous SAT [39].

### Subcutaneous SAT

**Figure 5:**
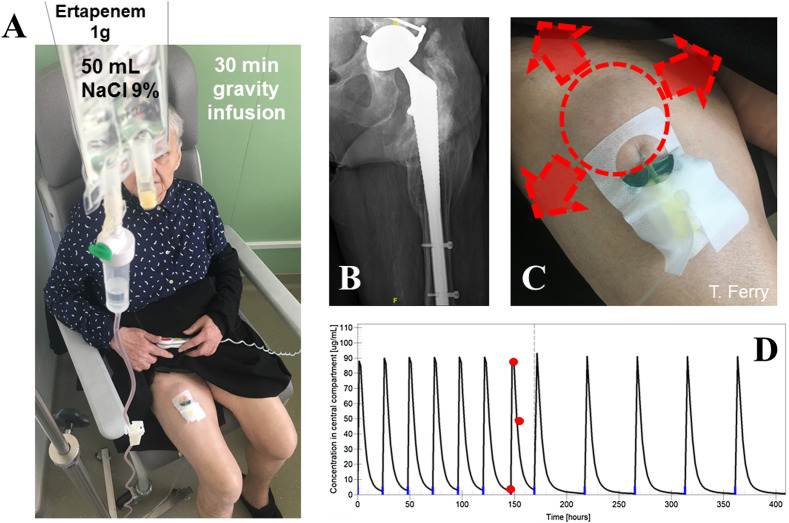
Example of dosage individualization based on PK/PD in a patient treated with ertapenem as SAT for a relapsing PJI: 78-old woman who had a relapsing left prosthetic hip infection due to *Enterobacter cloacae* (only susceptible to ertapenem with a MIC of 0.064 mg/L) for whom iterative DAIR was done with persistence of the organism (panel A). Explantation was contraindicated as it was a revision prosthesis without loosening with a high risk of peroperative complication and loss of function (panel B). She received as primary antibiotics following the DAIR ertapenem at the conventional dose of 1 g/day. Subcutaneous administrations were firstly performed using a butterfly needle with injection each day in the right thigh of in the abdominal flanks (panel C), with secondary systemic diffusion of the drug in blood, and then at the site of infection. Ertapenem drug concentrations were measured, with three samples collected in red (panel D; the *x*-axis shows the time, the *y* axis represents the ertapenem plasma concentration at the steady-state; The blue marks on the *x*-axis show drug administrations): pre-dose, 30 min after the end of the 30 min SC infusion and 5 h post-dose. Ertapenem individual PK parameters were then estimated by a Bayesian approach based on our published population PK model of ertapenem implemented into the BestDose™ software. Panel D shows the results of the model fitting (black line for estimated concentrations during time), which was very good, and we simulated a future regimen with 1 g of ertapenem every 48 h. Our plasma concentration target for this patient was the bacterial MIC (0.064 mg/L) corrected for ertapenem protein binding (free fraction of 5%), resulting in 1.28 mg/L. We calculated that 1 g of ertapenem every 48 h would result in a trough concentration of 0.4 mg/L and 63% of time spent above the target after 48 h. While the optimal value would be 100%, this was considered acceptable, considering that 40% was reported to be sufficient to get a bactericidal effect with this agent [54].

Suppressive subcutaneous antimicrobial therapy is an emergent way of SAT from our group [39]. In case of infections caused by Gram-negative pathogens, few oral options are available, especially if the pathogen is resistant to fluroquinolones and cotrimoxazole. In these patients, it is not possible to imagine intravenous SAT, due to the high risk of catheter complications. We developed since many years the off-label use of ceftriaxone, ceftazidime, and ertapenem by the subcutaneous route, in a concept called outpatient subcutaneous antimicrobial therapy (OSCAT) [39]. As compassionate treatment, we proposed to some patient the OSCAT as SAT. For that purpose, we developed a pharmacokinetic/pharmacodynamic (PK/PD) approach for dosage individualization of SAT by OSCAT in patients with BJI. In case of SAT with injectable beta-lactams, important questions are the dosage regimen that should be administered. Conventional dosing of beta-lactams is based on daily (ceftriaxone or ertapenem) or multiple daily intravenous administrations (ceftazidime), depending on the half-life of the molecules used. Spacing drug administration is desirable in patients with SAC, but it should respect the PK/PD targets to ensure treatment efficacy. Otherwise, the subcutaneous (SC) route may facilitate drug administration in patients with poor venous access, while preserving the PK/PD objectives of beta-lactams [40]. Therapeutic drug monitoring of the drug is first performed under conventional dosing, before considering the dose for SAT, as performed in an illustrative case (Figure 5). The results are analyzed by PK modeling which permits to estimate the patients’ individual PK parameters (e.g., clearance, volume of distribution) and simulate the future dosing regimen for SAT. Future dosing regimens with increased dosing interval (e.g., every 48 h, or three administrations per week) are examined using a Bayesian approach based on our published population PK model of ertapenem implemented into the BestDose™ software. This method is routinely applied in patients requiring ceftriaxone, ertapeneme of ceftazidime as SAT in our center, leading most of time in only one SC administration of the drug every 48 h, without occurrence of adverse event at the injection site. This approach is clearly acceptable for most of patients, with a clear benefit/risk to keep the function.

As the risk of relapse remains possible under SAT, we imagined some innovative intervention to act on directly on the biofilm, during the DAIR procedure, to facilitate the success of SAT.

## Phage therapy

**Figure 6:**
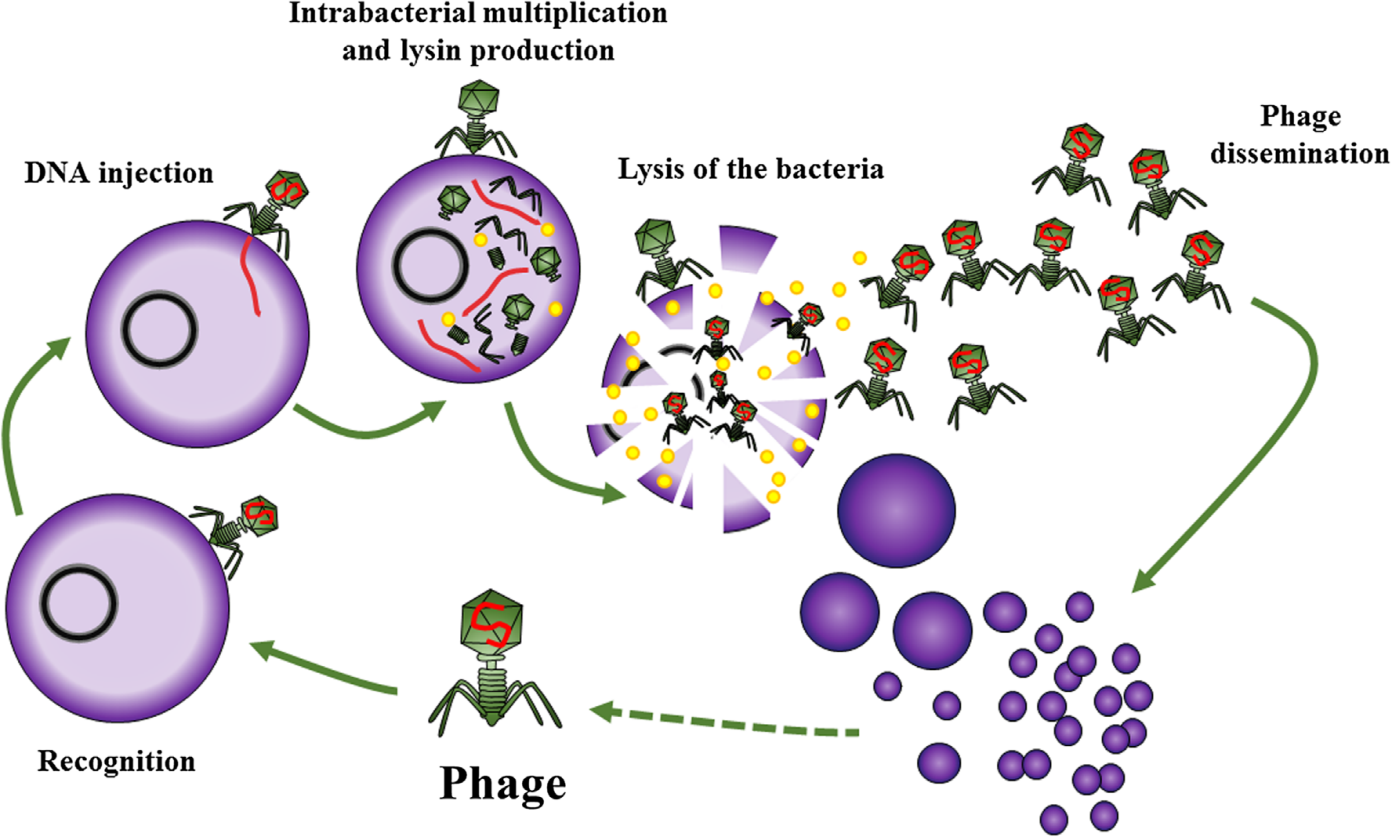
A phage and its lytic circle: firstly, the phage recognizes the specific bacteria, and injects its genetic material. The phage replicates itself into the bacterial cells by hacking the bacterial replication system and produces a lysin that induces a bacterial cell wall rupture, thus freeing hundreds of new phagic components that can in their turn target other bacterial cells located in the close environment in an exponential and self-sustained reaction.

**Figure 7:**
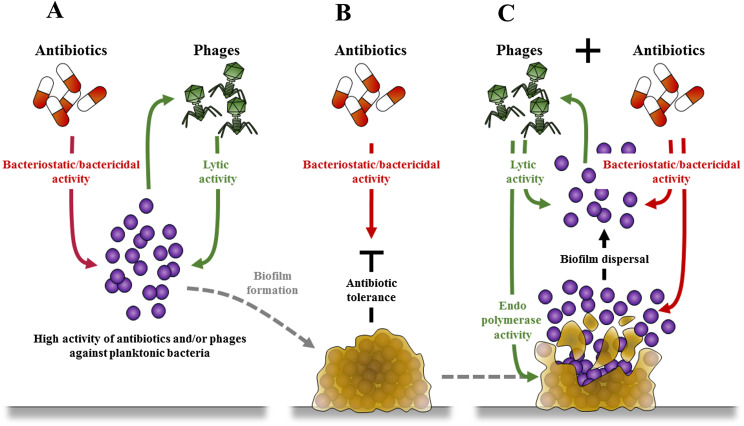
Activity of antibiotics alone or in combination on planktonic bacteria and on bacteria embedded in biofilm at a PJI surface: Antibiotics are active on planktonic bacteria, but not on bacteria embedded in biofilm. The phages are active on planktonic bacteria, and have the ability to replicate themselves among planktonic bacteria. The phage has the ability to disrupt the biofilm and acts synergistically with antibiotics to kill the bacteria in the biofilm and the bacteria released by the biofilm. In addition, synergistic effects are observed when phages are combined with antibiotics.

Bacteriophages are natural viruses that target bacteria. They have high environmental prevalence, especially in aqueous media such as salt or fresh water, drains, and soil. They seem to play a major role in bacteriological ecology in nature. Each is specific to one bacterial species. For instance a bacteriophage that target *Pseudomonas aeruginosa*, will only have the possibility to target this pathogen, and not another one. Some bacteriophages have a lytic cycle, leading to the destruction of the bacteria (Figure 6). These bacteriophages have the ability to hijack the bacterial machinery to produce hundreds of virions, with lysis of the host by production of a lysin (also called endolysin) that disrupts the bacterial cell wall, allowing the release of progeny virions from the lysed bacteria. This can give rise to an exponential self-maintaining phenomenon, with the virions replicating until nothing is left of the host (Figure 6). Phage therapy consists in using lytic phages to treat bacterial infection. The concept was first described by the French microbiologist Félix d’Hérelle (1873–1949). Working in the Institut Pasteur in Paris, in 1917 he showed that patients who recovered from dysentery implicating *Shigella* had phages with specific activity against *Shigella* in their stool. Phage therapies were later developed against pathogens responsible for digestive infections, and for skin infection such as streptococci and staphylococci. The phages were produced in the Bacteriophage private laboratory, founded by d’Hérelle in France, and in Tbilisi (Georgia), where he founded the Eliava Institute with his student Georges Eliava. This center is still open and hundreds of patients from the west go to this center to receive phages, mainly for the following indications: cystic fibrosis, relapsing urinary tract infection, and osteomyelitis. Phages were also produced and used in Lyon in the 1960s, before being sidelined due to the large availability of multiple oral and intravenous antibiotics with a large spectrum of action [41]. More recently, Patey et al. in the Villeneuve Saint Georges hospital in France, reported their experience with phage therapy in 15 French patients between 2006 and 2018, using phages produced in Russia or in the Eliava Institute. Nine of the patients had resistant bone and joint infection, including post-traumatic osteomyelitis and implant infection. Phages were administered intraoperatively and postoperatively on exposed bone or injected into the surgical drains. Some clinical success was reported, but the BJIs and the medical and surgical treatments were very heterogeneous [42]. More recently, Tkhilaishvili et al. reported a case of chronic relapsing PJI due to multidrug-resistant *P. aeruginosa*. They performed prosthesis explantation, with local application of anti-*P. aeruginosa* bacteriophages from the Georgian collection during surgery and each eight hours during five days by using drainages tubes [43]. In Belgium, the Queen Astrid military hospital (QAMH) in Brussels developed a production unit to treat patients with complex bacterial infections as compassionate treatment, but there is no orthopedic unit to manage the patient in this institution. Few private companies in the world (in USA, France, Austria) try to purify bacteriophages to perform clinical trial and finally obtain authorization for a clinical use from health authorities [44, 45]. Indeed, in France, it is not allowed to import and use the Georgian or Russian phages, as their production does not fulfill the criteria of the “good manufacturing practice” (GMP) guidelines and they may contain pyogenic substances (endotoxins or other bacterial debris). A French private company has produced purified phages and performed a clinical trial in Europe called Phagoburn [46]. We developed the “PhagoDAIR” concept in our institution, by injecting during open or arthroscopic DAIR, a single shot of a cocktail of active bacteriophages, after multidisciplinary and ethical discussions, under supervision of French National Agency for Medicines and Health Products Safety (ANSM). There is a high rationale for the use of bacteriophages in patients with PJI. First of all, they are active on planktonic bacteria, as antibiotics (Figure 7, panel A). Contrary to antibiotics that are not active on bacteria embedded in biofilm (Figure 7, panel B), phages have the ability to disrupt biofilm facilitating the efficacy of antibiotics that could be prescribed in the same time [47, 48]. Based on these pathophysiological elements, we treated up to now ~10 patients with relapsing PJI despite a previous DAIR followed by SAT, mainly patients with revision prosthesis without loosening, with a good clinical response to the phage injections during DAIR. These patients keep their function with disappearance of clinical signs of infection [41, 49].

## Phage lysins

Lysins are hydrolytic enzymes produced during the final stage of the lytic cycle by bacteriophages in order to cleave the bacterial cell wall (Figure 6). They are highly evolved enzymes that target the cell wall of the bacteria [50]. Phage lysins are generally species or subspecies specific, with a broader spectrum of action in comparison with bacteriophages. For instance, bacteriophages targeting *S. aureus* are usually not active against coagulase-negative staphylococci, whereas the lysin purified from these bacteriophages are active against *S. aureus*, but also against coagulase-negative staphylococci. Some companies are developing recombinant lysins and CF-301 (Exebacase) is the most advanced staphylococcal lysin in development [50, 51]. The production and development processes of lysins follow the classical approval ways as any drugs. CF-301 has a synergistic antimicrobial activity and has been shown to be highly effective in clearing biofilms in different models [50–53]. We treated as compassionate therapy some patients with relapsing multidrug-resistant *S. epidermidis* PJI for whom no phages were available by arthroscopic DAIR with one shot administration of CF-301 into the joint at the end of the procedure, followed by SAT, with interesting results in some patients.

## Conclusion

It is crucial to understand the pathophysiology of BJI to imagine particular anti-infectious interventions that could facilitate the infectious control, to keep the function. Indeed, in patient with infected revision prosthesis who has still a good function without prosthesis loosening, prosthesis exchange has to be avoided or delayed, to limit dramatic and definitive loss of function. This type of concept could only emerge in excellence center such as CRIOAc by considering the pathophysiology of the disease and the different parameters that have to be reported and shared by orthopedic surgeons, infectiologists, and microbiologists managing together the patient, and the research. The next step will be to evaluate such practices in nation-wide clinical trials.
